# Studying behavior under constrained movement

**DOI:** 10.7554/eLife.91145

**Published:** 2023-08-30

**Authors:** Ranier Gutierrez

**Affiliations:** 1 Laboratory of Neurobiology and Appetite, Department of Pharmacology, CINVESTAV Mexico City Mexico

**Keywords:** reward, motivation, ingestive behavior, mouse, neuroscience tools, Mouse

## Abstract

A new platform for studying how brain activity is linked to behavior enables researchers to perform diverse experiments on mice that have their heads immobilized.

**Related research article** Gordon-Fennell A, Barbakh JM, Utley MT, Singh S, Bazzino P, Gowrishankar R, Bruchas MR, Roitman MF, Stuber GD. 2023. An open-source platform for head-fixed operant and consummatory behavior. *eLife* **12**:e86183. doi: 10.7554/eLife.86183.

A central question in neuroscience is how patterns of brain activity lead to different behaviors. There is growing recognition that the brain is an active organ that constantly anticipates, predicts, and interacts with its environment (Buzsaki, 2021). It is therefore important to study the brain and behavior of animals in as natural conditions as possible. However, the more ‘free’ an animal is to behave as it would in the wild, the harder it is to observe what the brain is doing.

To study how brain activity is linked to behavior, researchers often immobilize the heads of experimental animals via a technique known as head fixation (Guo et al., 2014; Hughes et al., 2020). The approach has made it possible to observe and measure brain activity in fully conscious animals that can still move other parts of their body, such as their tongue, eyes, and limbs. It also allows scientists to perform experiments that would be impossible to conduct on non-constrained animals, such as using external tools to stimulate the activity of certain nerves or parts of the body (Guo et al., 2014). This has led to the development of sophisticated recording techniques to monitor brain activity during behavioral tasks that isolate specific functions of the brain.

Although this technique was initially used on primates (Evarts, 1968), recent advances in technology have made it possible to record neural activity in the brains of head-fixed mice (Allen et al., 2019; Marshel et al., 2019; Musall et al., 2019; Sofroniew et al., 2016; Steinmetz et al., 2019; Stringer et al., 2019). A recent study of head-fixed mice by a consortium of 11 labs found that almost any behavioral task elicits brain-wide responses that are more related to movement than to sensory information or cognitive variables such as memory or decision making (Benson et al., 2023; Musall et al., 2019). This suggests that even during head fixation, body movement – particularly licking behavior (Gutierrez et al., 2010; Benson et al., 2023) – still has an important impact on brain activity, making it a useful tool for neuroscience studies. Despite the benefits head fixation provides, many of the tasks used to study behavior in freely moving animals are difficult to carry out on head-fixed rodents.

Now, in eLife, Garret Stuber and colleagues from the University of Washington and University of Illinois at Chicago – including Adam Gordon-Fennell as first author – report a new low-cost system for conducting behavioral experiments related to motivation on head-fixed mice (Gordon-Fennell et al., 2023). The tool – which is named OHRBETS (short for Open-Source Head-fixed Rodent Behavioral Experimental Training System) – consists of two configurations (Figure 1). The first is used to study operant conditioning, a process where behaviors change in response to either positive or negative reinforcement (Figure 1A). In this set-up, a spout delivers rewards, such as sucrose, to mice when they turn a wheel to the left or right. The device also allows specific movements made by the mice, such as turning the wheel, to self-activate certain brain cells associated with either positive (delivery of pleasant stimuli) or negative (termination of an undesirable stimulus) reinforcement. Finally, the device can measure real-time place preference behavior, where turning the wheel triggers self-stimulation of different brain cells that results in a rewarding or aversive experience.

**Figure 1.**
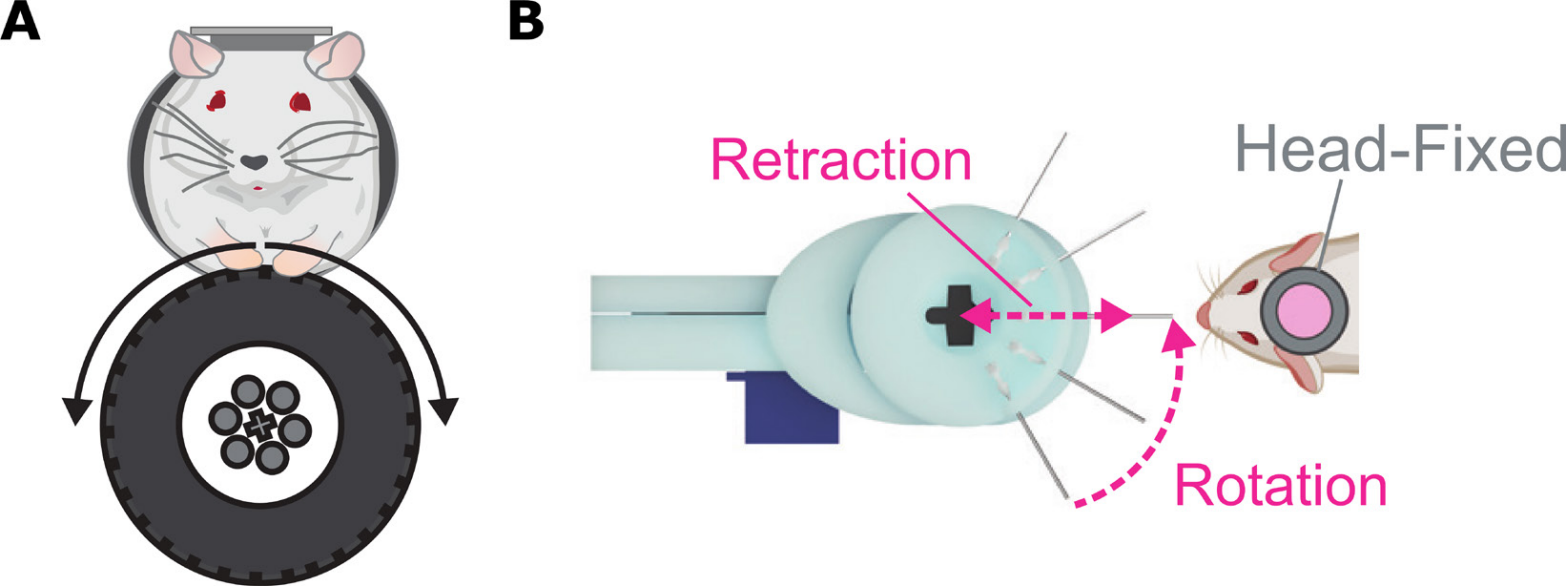
A new platform for studying behaviors related to motivation in head-fixed mice. (**A**) Operant configuration: mice are trained to rotate a wheel to the left or right to obtain sucrose, to trigger stimulation of certain brain areas, or to express real-time preference and avoidance behavior. (**B**) Consummatory configuration: multiple spouts (represented as lines) carrying solutions with varying levels of sucrose are consecutively rotated in front of the head-fixed mouse for them to consume. The brain activity, release of neurochemicals, and behavior of the mice is then recorded to see how they respond to these different tasting solutions.

Image credit: Adapted from Figures 2F and 5A in Gordon-Fennell et al., 2023

The second configuration is used to investigate behaviors linked to consuming solutions which produce taste sensations, known as tastants (Figure 1B). It contains multiple spouts which are loaded with varying concentrations of sucrose. This allows researchers to assess how mice respond to different tastes (measured by how often they lick the spout), similar to a behavioral task used to study palatability in freely moving rodents (Smith, 2001).

In a behavioral tour de force, Gordon-Fennell et al. found that head-fixed mice tested with OHRBETS under these different conditions displayed behavior that is comparable to that observed in freely moving mice. By replicating various tasks used in freely moving systems in a head-fixed setup, researchers can take advantage of the precise measurements and reproducibility of the head-fixed set-up while retaining some of the natural behavioral characteristics observed in freely moving experiments.

While OHRBETS increases the number of behavioral tasks that can be evaluated in head-fixed mice, it is important to highlight that constrained settings also have some disadvantages. One important limitation is that it can cause discomfort, potentially putting the animal under stress which can lead to changes in brain activity (Juczewski et al., 2020). Furthermore, most experiments currently carried out in neuroscience laboratories, including those conducted on freely moving rodents, are far from natural. They are usually performed during daylight hours (despite rodents being nocturnal), and in environments lacking sensory stimuli that would be found in the wild, such as food to forage or other animals to interact with. This underscores the challenges ahead and the urgent need to develop new methods to continuously record brain activity and monitor behavior (day and night) with minimal human intervention. Despite these limitations, OHRBETS is a powerful tool for accelerating understanding of how the brain functions under constrained movement and will provide a foundation for understanding how the brain works in more natural settings in the future.

## References

[bib1] Allen WE, Chen MZ, Pichamoorthy N, Tien RH, Pachitariu M, Luo L, Deisseroth K (2019). Thirst regulates motivated behavior through modulation of brainwide neural population dynamics. Science.

[bib2] Benson B, Benson J, Birman D, Bonacchi N, Carandini M, Catarino JA, Chapuis GA, Churchland AK, Dan Y, Dayan P, DeWitt EE, Engel TA, Fabbri M, Faulkner M, Fiete IR, Findling C, Freitas-Silva L, Gerçek B, Harris KD, Häusser M, Hofer SB, Hu F, Hubert F, Huntenburg JM, Khanal A, Krasniak C, Langdon C, Lau PYP, Mainen ZF, Meijer GT, Miska NJ, Mrsic-Flogel TD, Noel JP, Nylund K, Pan-Vazquez A, Pouget A, Rossant C, Roth N, Schaeffer R, Schartner M, Shi Y, Socha KZ, Steinmetz NA, Svoboda K, Urai AE, Wells MJ, West SJ, Whiteway MR, Winter O, Witten IB, International Brain Laboratory (2023). A Brain-Wide Map of Neural Activity during Complex Behaviour. bioRxiv.

[bib3] Buzsaki G (2021). The Brain from Inside Out.

[bib4] Evarts EV (1968). Relation of pyramidal tract activity to force exerted during voluntary movement. Journal of Neurophysiology.

[bib5] Gordon-Fennell A, Barbakh JM, Utley MT, Singh S, Bazzino P, Gowrishankar R, Bruchas MR, Roitman MF, Stuber GD (2023). An open-source platform for head-fixed operant and consummatory behavior. eLife.

[bib6] Guo ZV, Hires SA, Li N, O’Connor DH, Komiyama T, Ophir E, Huber D, Bonardi C, Morandell K, Gutnisky D, Peron S, Xu N, Cox J, Svoboda K (2014). Procedures for behavioral experiments in head-fixed mice. PLOS ONE.

[bib7] Gutierrez R, Simon SA, Nicolelis MAL (2010). Licking-induced synchrony in the taste-reward circuit improves cue discrimination during learning. The Journal of Neuroscience.

[bib8] Hughes RN, Bakhurin KI, Barter JW, Zhang J, Yin HH (2020). A head-fixation system for continuous monitoring of force generated during behavior. Frontiers in Integrative Neuroscience.

[bib9] Juczewski K, Koussa JA, Kesner AJ, Lee JO, Lovinger DM (2020). Stress and behavioral correlates in the head-fixed method: stress measurements, habituation dynamics, locomotion, and motor-skill learning in mice. Scientific Reports.

[bib10] Marshel JH, Kim YS, Machado TA, Quirin S, Benson B, Kadmon J, Raja C, Chibukhchyan A, Ramakrishnan C, Inoue M, Shane JC, McKnight DJ, Yoshizawa S, Kato HE, Ganguli S, Deisseroth K (2019). Cortical layer-specific critical dynamics triggering perception. Science.

[bib11] Musall S, Kaufman MT, Juavinett AL, Gluf S, Churchland AK (2019). Single-trial neural dynamics are dominated by richly varied movements. Nature Neuroscience.

[bib12] Smith JC (2001). The history of the “Davis Rig.”. Appetite.

[bib13] Sofroniew NJ, Flickinger D, King J, Svoboda K (2016). A large field of view two-photon mesoscope with subcellular resolution for in vivo imaging. eLife.

[bib14] Steinmetz NA, Zatka-Haas P, Carandini M, Harris KD (2019). Distributed coding of choice, action and engagement across the mouse brain. Nature.

[bib15] Stringer C, Pachitariu M, Steinmetz N, Reddy CB, Carandini M, Harris KD (2019). Spontaneous behaviors drive multidimensional, brainwide activity. Science.

